# Interplay between pairing and correlations in spin-polarized bound states

**DOI:** 10.3762/bjnano.9.129

**Published:** 2018-05-07

**Authors:** Szczepan Głodzik, Aksel Kobiałka, Anna Gorczyca-Goraj, Andrzej Ptok, Grzegorz Górski, Maciej M Maśka, Tadeusz Domański

**Affiliations:** 1 Institute of Physics, M. Curie-Skłodowska University, 20-031 Lublin, Poland; 2 Institute of Physics, University of Silesia, 41-500 Chorzów, Poland; 3 Institute of Nuclear Physics, Polish Academy of Sciences, 31-342 Kraków, Poland; 4 Faculty of Mathematics and Natural Sciences, University of Rzeszów, 35-310 Rzeszów, Poland

**Keywords:** bound states in superconductors, Majorana quasiparticles, subgap Kondo effect

## Abstract

We investigate single and multiple defects embedded in a superconducting host, studying the interplay between the proximity-induced pairing and interactions. We explore the influence of the spin–orbit coupling on energies, polarization and spatial patterns of the bound (Yu–Shiba–Rusinov) states of magnetic impurities in a two-dimensional square lattice. We also address the peculiar bound states in the proximitized Rashba chain, resembling the Majorana quasiparticles, focusing on their magnetic polarization that has been recently reported by S. Jeon et al. (*Science ***2017, ***358,* 772). Finally, we study leakage of these polarized Majorana quasiparticles into side-attached nanoscopic regions and confront them with the subgap Kondo effect near to the singlet–doublet phase transition.

## Introduction

Magnetism is usually detrimental to superconductivity because it breaks the Cooper pairs (at the critical field strength *H_c2_*). There are, however, a few exceptions in which these phenomena coexist, e.g., in iron pnictides [1], CeCoIn_5_ [2]. Also, sometimes magnetic fields induce superconductivity [3]. Plenty of other interesting examples can be found in nanoscopic systems, where magnetic impurities (dots) exhibit a more subtle relationship with the electron pairing driven by the proximity effect [4–5]. Cooper pairs easily penetrate the nanoscopic impurities, inducing the bound (Yu–Shiba–Rusinov) states that manifest the local pairing in coexistence with magnetic polarization. Such bound states have been observed in various systems [6–14]. In-gap states (appearing in pairs symmetrically around the Fermi level) can be nowadays controlled electrostatically or magnetically [12] whereas their topography, spatial extent and polarization can be precisely inspected by the state-of-art tunneling measurements [15–16].

It has been reported that adatoms deposited on a two-dimensional (2D) superconducting surface develop Yu–Shiba–Rusinov (YSR) states, extending to a dozen of intersite distances and they reveal particle–hole oscillations [11]. Bound states of these magnetic impurities in superconducting NbSe_2_ are characterized by the star shape [17] typical for the rotational symmetry of its triangular lattice. More complex objects, such as dimers, reveal other spatial features, showing the bonding and antibonding states [18]. In a somewhat different context it has been pointed out [19] that exchange coupling between numerous quantum defects involving their intrinsic spins can couple them ferromagnetically. This can be used (e.g., in metallic carbon nanotubes) for a robust transmission of magnetic information over large distances.

In all cases the bound YSR states are also sensitive to interactions. One of them is the spin–orbit coupling (usually meaningful at boundaries, e.g., surfaces) [20–22]. Such interaction in one-dimensional magnetic nanowires can induce the topologically nontrivial superconducting phase, in which the YSR states undergo mutation to Majorana (zero-energy) quasiparticles. Coulomb repulsion between the opposite spin electrons can bring additional important effects. In the proximitized quantum dots it can lead to a parity change (quantum phase transition) with further influence on the subgap Kondo effect (driven by effective spin-exchange coupling with mobile electrons). Furthermore, such spin exchange can be amplified by the induced electron pairing, and can have constructive influence on the Kondo effect [23–24].

We study here the polarized bound states, taking into account the spin–orbit and/or Coulomb interactions. In particular, we consider: (i) a single magnetic impurity in a 2D square lattice of a superconducting host, (ii) a nanoscopic chain of magnetic impurities on the classical superconductor (i.e., proximitized Rashba nanowire) in its topologically trivial/nontrivial superconducting phase, and (iii) a strongly correlated quantum dot side-attached to the Rashba chain, where the Kondo and the leaking Majorana quasiparticle can be confronted with each other. These magnetically polarized YSR and Majorana quasiparticles as well as the subgap Kondo effect can be experimentally verified using tunneling heterostructures with ferromagnetic lead (STM tip).

## Results and Discussion

### Single magnetic impurity

Let us start by considering a single magnetic impurity on the surface of an *s*-wave superconductor in presence of spin–orbit interactions. This situation can be modeled by the Anderson-type Hamiltonian

[1]
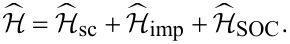


We describe the superconducting substrate by

[2]
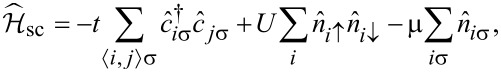


where 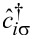 (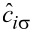) denotes creation (annihilation) of an electron with spin σ at the *i*-th site, *t* is a hopping integral between the nearest neighbors, μ is the chemical potential, and 
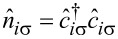
 is the number operator. For simplicity, we assume a weak attractive potential *U <* 0 between itinerant electrons and treat it within the mean-field decoupling


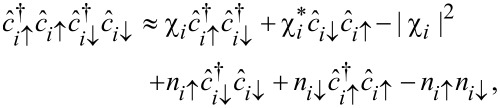


where 
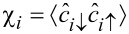
 is the local superconducting order parameter and *n_iσ_* = 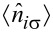. The Hartree term can be incorporated into the local (spin-dependent) chemical potential μ → 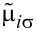 ≡ μ − 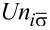. The second term in Equation 1 refers to the local impurity

[3]
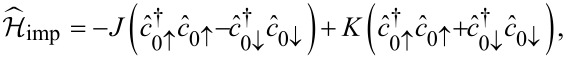


which affects the order parameter χ*_i_* near the impurity site *i* = 0, inducing the YSR states [25–26]. In this work we focus on the magnetic term *J* [4,27], disregarding the potential scattering *K*.

The spin–orbit coupling (SOC) can be expressed by

[4]
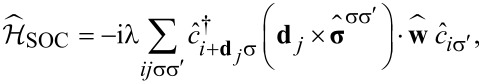


where the vector 
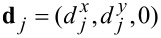
 refers to positions of the nearest neighbors of the *i*-th site, and 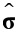 = (σ*_x_*, σ*_y_*, σ*_z_*) stands for the Pauli matrices. The unit vector 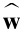 shows the direction of the spin–orbit field, which can be arbitrary. Here we restrict our considerations to the in-plane 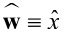 = (1, 0, 0) polarization, which will be important for nontrivial superconductivity in nanowires discussed in the subsection ’Magnetically polarized Majorana quasiparticles’. The other (out-of-plane) component could eventually mix ↑ and ↓ spins [22].

Impurities break the translational invariance, therefore the pairing amplitude χ*_i_* and occupancy *n_i_*_σ_ have to be determined for each lattice site individually. We can diagonalize the Hamiltonian (Equation 1) by the unitary transformation

[5]
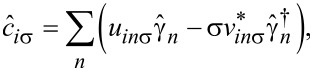


where 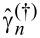 are quasiparticle fermionic operators with eigenvectors *u_in_*_σ_ and *v_in_*_σ_. This leads to the Bogoliubov–de Gennes (BdG) equations

[6]
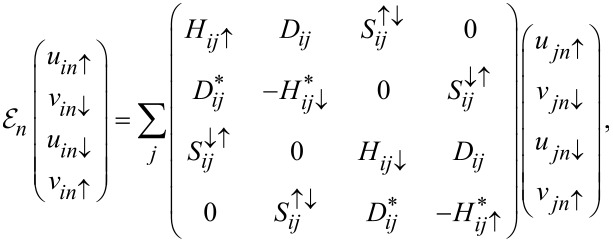


where *D_ij_* = δ*_ij_U*χ*_i_*, and the single-particle term is given by


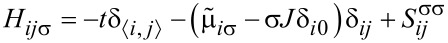


with the spin–orbit coupling term


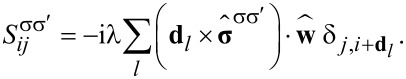


Here, 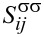 and 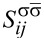 (where 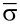 is opposite to σ) correspond to in-plane and out-of-plane spin–orbit field, respectively, and satisfy 
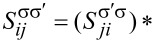
.

Solving numerically the BdG equations (Equation 6) we can determine the local order parameter χ*_i_* and occupancy *n_i_*_σ_

[7]
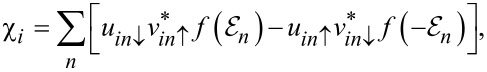


[8]
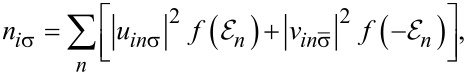


where *f*(ω) = [1 + exp(ω/*k*_B_*T*)]^−1^. In what follows, we shall inspect the spin-resolved local density of states


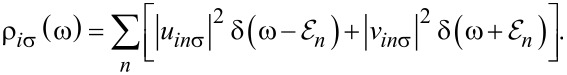


For its numerical computation we replace the Dirac delta function with the Lorentzian function δ(ω) = ζ/[π(ω^2^ + ζ^2^)] with a small broadening ζ = 0.01 t. We have solved the BdG equations, considering a single magnetic impurity in a square lattice, comprising *N_a_* × *N_b_* = 41 × 41 sites. We assumed *U*/*t* = −3, μ/*t* = 0, and determined the bound states for two representative values of the spin–orbit coupling λ upon varying *J*.

**Figure 1:**
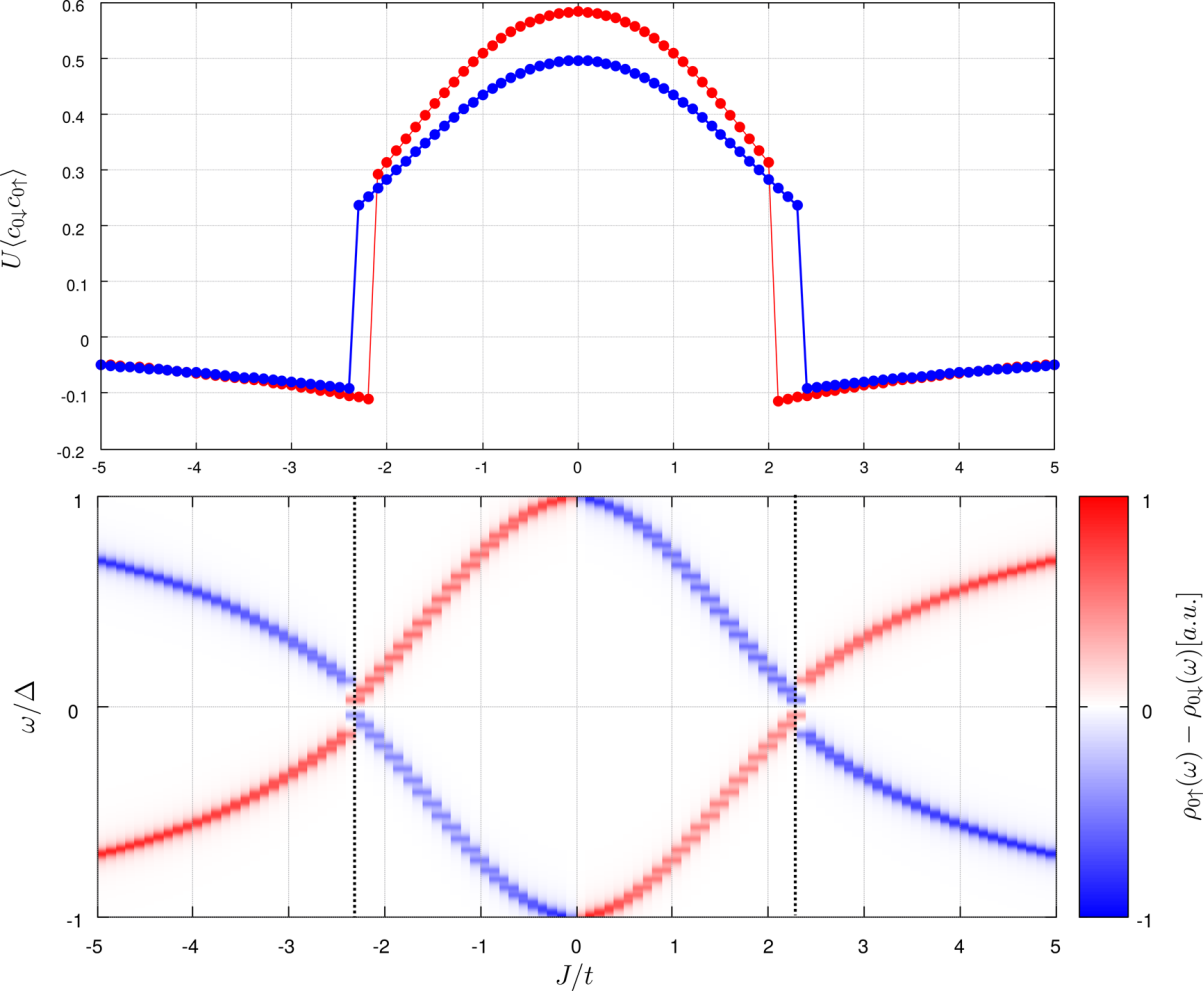
The local order parameter obtained at zero temperature for weak λ/*t* = 0.1 (red line) and strong spin–orbit coupling λ/*t* = 1 (blue line). The bottom panel shows the energies and magnetic polarization ρ_0↑_(ω) − ρ_0↓_(ω) of YSR states obtained in the weak-coupling limit λ/*t* = 0.1.

The magnetic potential has substantial influence on the local order parameter χ_0_. In particular, at some critical value *J*_c_ this quantity discontinuously changes its magnitude and sign (see the upper panel in Figure 1), signaling a first-order phase transition [28–30]. This quantum phase transition at *J*_c_ is an artifact of the classical spin approximation. When spin fluctuations are allowed, a Kondo-like crossover is obtained instead of a first-order phase transition [31–32]. In general, the quasiparticle spectrum at the impurity site is characterized by two bound states ±*E*_YSR_ inside the gap Δ of the superconducting host (displayed in the bottom panel of Figure 1). These energies ±*E*_YSR_ and the related spectral weights depend on *J*. At *J* = *J*_c_ the YSR bound states cross each other *E*_YSR_(*J*_c_) = 0 and their crossing signifies the ground-state parity change [33] from BCS-type (spinless) to the singly occupied (spinful) configurations [8,15,21,34]. Let us remark that this quantum phase transition is also accompanied with a reversal of the YSR polarization (see bottom panel in Figure 1). A similar behavior can be observed also for multiple impurities, at several critical values of *J* [35].

**Figure 2:**
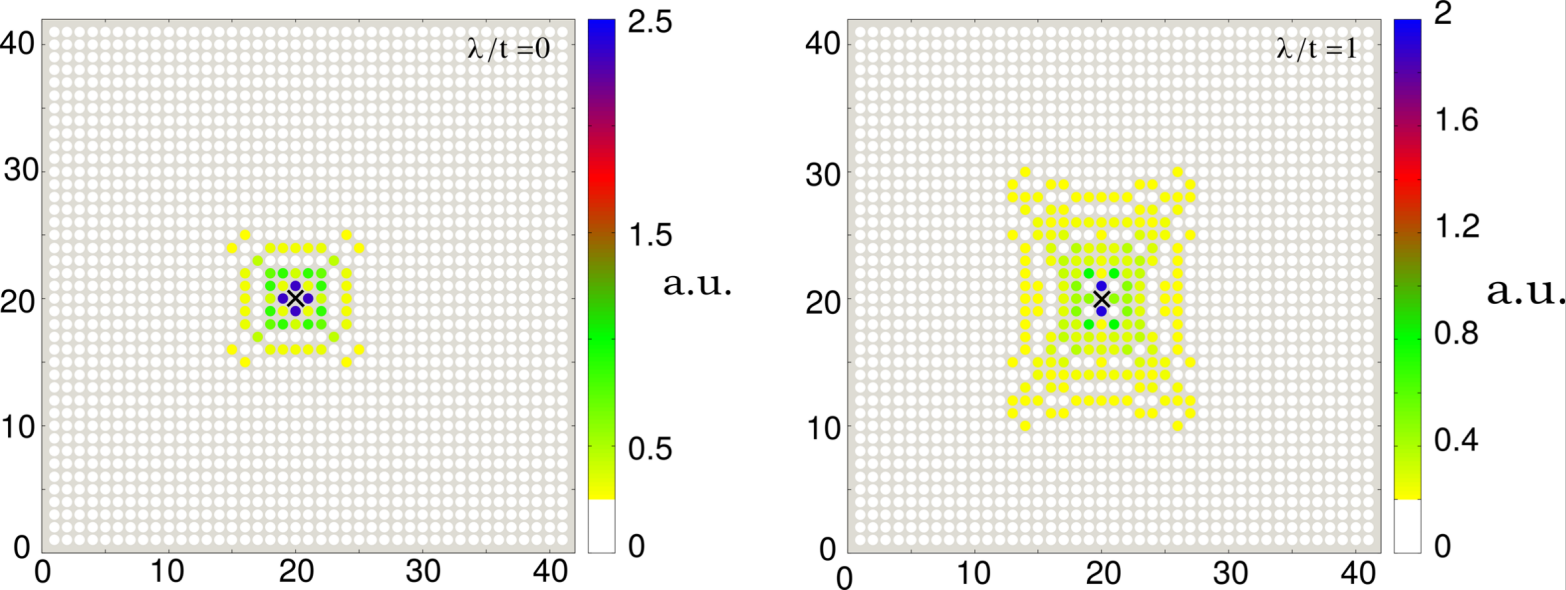
Spatial profiles of the YSR states 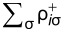 obtained for |*J*| *< J*_c_ in the absence of spin–orbit coupling (left panel) and for strong in-plane coupling λ = *t* (right panel). The spin–orbit field is chosen along the *x*-axis and leads to an additional imaginary hopping term along the *y*-axis, which elongates the YSR states in the *y*-direction. The impurity spin is oriented along the (0, 0, 1) direction.

Within the BdG approach we can inspect spatial profiles of the YSR states by integrating the spectral weights


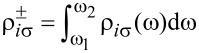


in the interval 
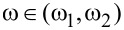
 capturing the quasiparticles at negative/positive energies ±*E*_YSR_ [36]. Figure 2 illustrates the results obtained for λ = 0 (left panel) and λ = *t* (right panel). We clearly notice a fourfold rotational symmetry (typical for the square lattice) and the spatial extent of YSR states reaching several sites away from the magnetic impurity. The non-vanishing difference of the spectral weight |*u_in_*_↑_|^2^ −|*u_in_*_↓_|^2^ at the positive energy ω = +*E_YSR_* and of |*v_in_*_↑_|^2^ −|*v_in_*_↓_|^2^ at the negative energy ω = −*E_YSR_* implies the effective spin-polarization of the bound states (their polarization is illustrated in the bottom panel of Figure 1).

**Figure 3:**
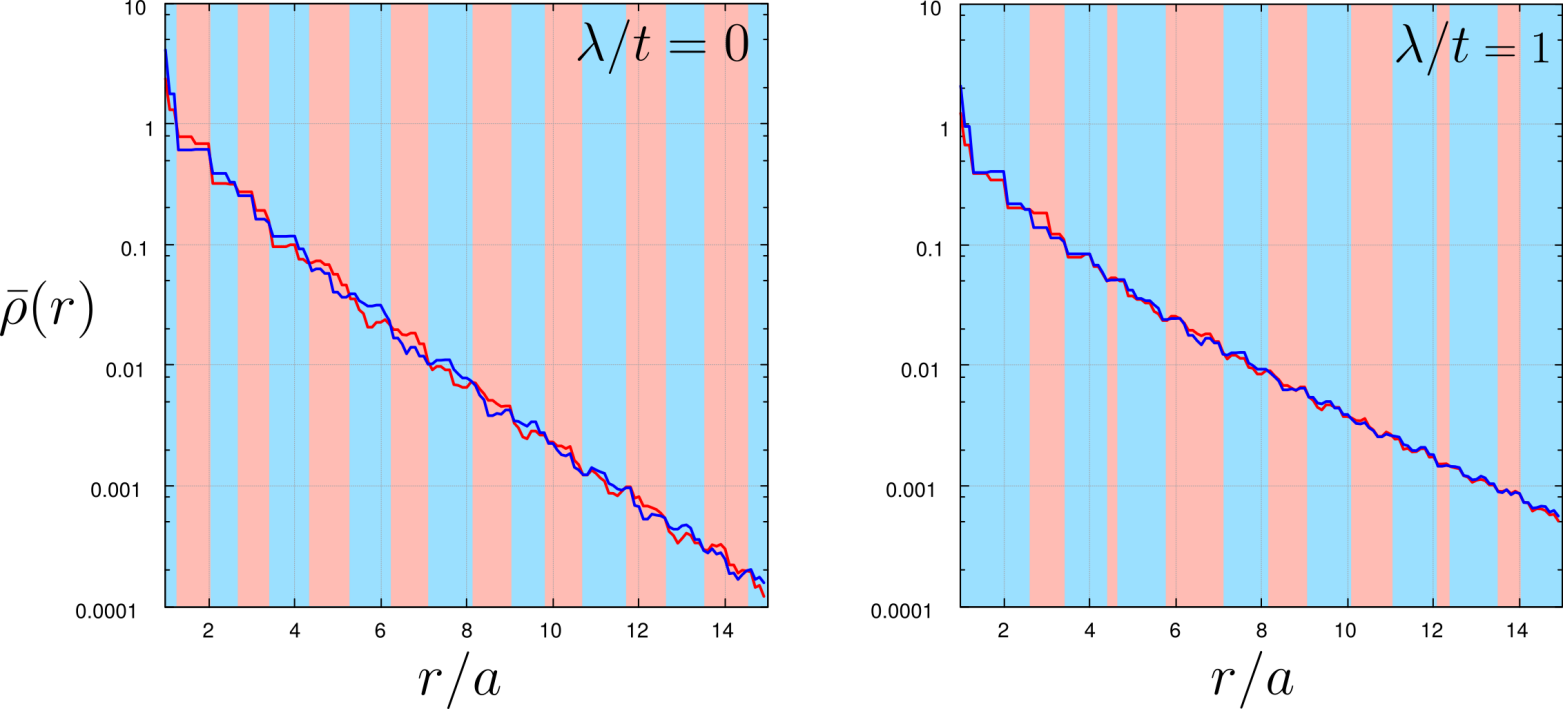
Hole-like (blue line) and electron-like (red line) displaced moving average 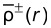 as a function of the radial distance *r* from the impurity site obtained for |*J*| *< J*_c_ using δ*r* = 0.5*a*. The blue and red background color indicates the dominant type (hole or particle) of the YSR states at a given distance *r*. The left and right panels correspond to λ = 0 and λ = *t*, respectively.

For a quantitative estimation of the spatially varying magnetization (driven by the particle–hole asymmetry) we have computed the displaced moving average 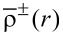, which corresponds to an averaged spectral weight contained in a ring of the radius *r* and a small half-width δ*r*. This quantity is sensitive only to the radial distance *r* from the magnetic impurity, averaging the angular anisotropy. Our results, presented in Figure 3, clearly indicate the spatial particle–hole oscillations 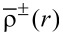 of the YSR states (compare the blue and red lines). Such particle–hole oscillations decay exponentially with *r* in agreement with previous studies [11,37–38]. The dominant (particle or hole) contributions to the YSR bound states are displayed by the alternating color of the background in Figure 3. We notice that the spin–orbit coupling seems to suppress these particle–hole oscillations.

Summarizing this section, we point out that the quantum phase transition at *J*_c_ depends on the spin–orbit coupling λ and it has experimentally observable consequences in the magnetization induced near the impurity site. For weak magnetic scattering |*J*| *< J*_c_ the impurity is partly screened, whereas for stronger couplings |*J*| *> J*_c_ the impurity polarizes its neighborhood in the direction of its own magnetic moment. Similar effects have been previously discussed in [21], but here we additionally consider the role of spin–orbit coupling. First of all, such interaction shifts the quantum phase transition (to larger values of *J*) and secondly it enhances the spatial extent of YSR states and gradually smoothes the particle–hole oscillations.

### Magnetically polarized Majorana quasiparticles

In this section we increase the number of impurities. Let us now imagine a nanoscopic chain of magnetic impurities (for instance Fe atoms) deposited on the surface of a conventional *s*-wave superconductor. We study the magnetically polarized bound states, focusing on the proximity-induced nontrivial superconducting phase. In practice, the quasiparticle spectrum can be probed within STM-type setups, by attaching a conducting [39–40], superconducting [41], or a magnetically polarized tip [42]. We assume the spin–orbit interaction aligned perpendicularly to the wire and the magnetic field parallel to it, leading to the effective intersite pairing of identical spins and (under specific conditions) inducing zero-energy end modes resembling Majorana quasiparticles. This issue has been recently studied very intensively but here we simply focus on the spin-polarized aspects of this problem.

Due to the spin–orbit interaction, momentum and spin are no longer “good” quantum numbers. By solving the problem numerically, however, we can estimate the percentage with which the true quasiparticles are represented by the initial spin. We have recently emphasized [43], that the amplitude of intersite pairing (between identical spin electrons) differs several times for ↑ and ↓ sectors. This leads to an obvious polarization of the YSR and Majorana quasiparticles (the latter appearing near the nanochain edges).

Let us consider the STM-type geometry relevant to the recent experimental situation addressed by A. Yazdani and co-workers [42], which can be described by the following Hamiltonian

[9]
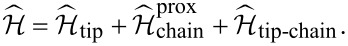


We assume here that the STM tip describes a polarized fermion gas


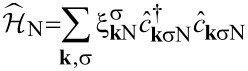


where the energy 
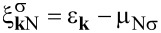
 can be controlled by some finite detuning of the chemical potentials μ_N↑_ − μ_N↓_. Individual atoms of the nanochain are coupled with such STM tip through


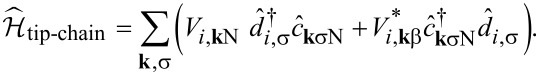


For simplicity, we assume constant couplings


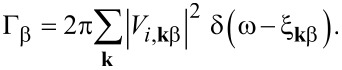


The low-energy physics of such proximitized Rashba nanowire can be described by [44]

[10]
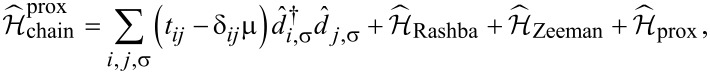


where 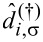 annihilates (creates) an electron of spin σ at site *i* with energy ε*_i_*, and *t_ij_* is the hopping integral. The effective intersite (*p*-wave) pairing is induced through a combined effect of the Rashba and the Zeeman terms

[11]
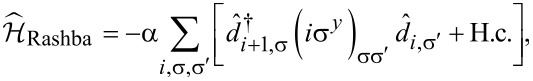


[12]
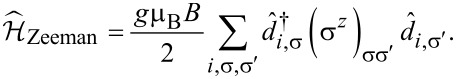


The proximity effect, which induces the on-site (trivial) pairing, can be modelled as [45]

[13]
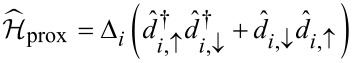


with the local pairing potential Δ*_i_* = Γ_S_/2.

**Figure 4:**
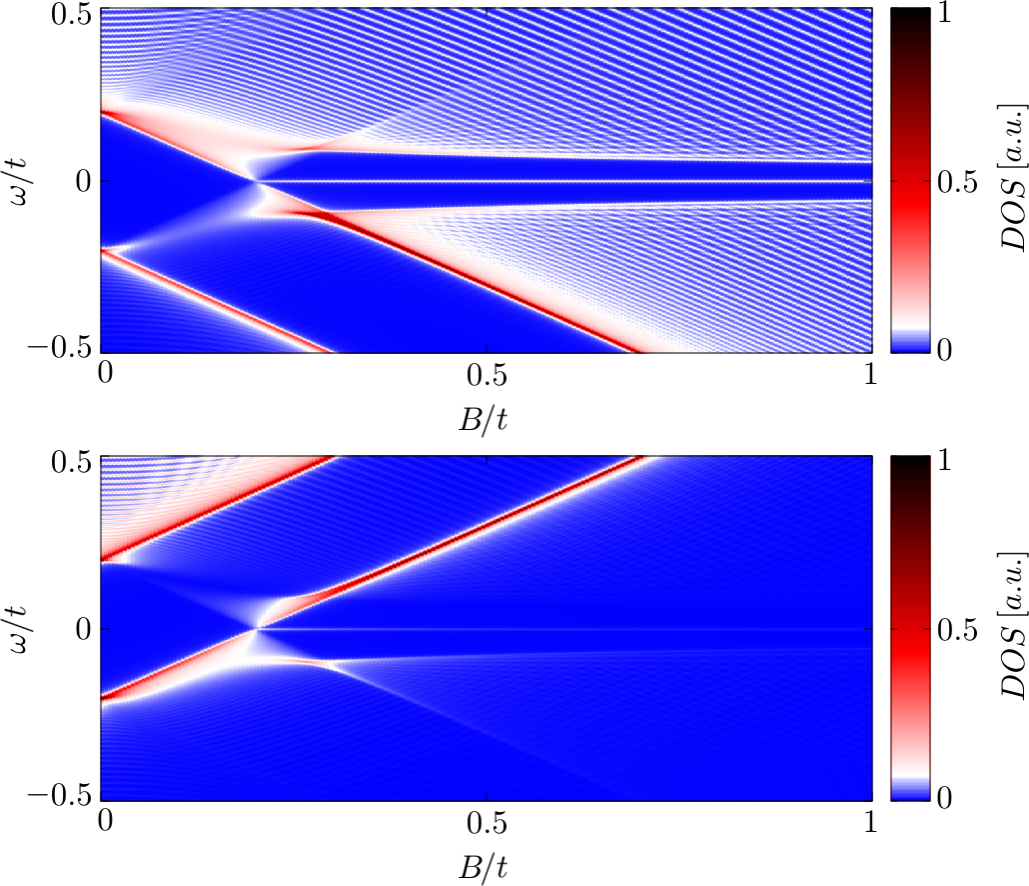
The effective quasiparticle spectrum ρ*_i_*_σ_(ω) as a function of a magnetic field *B* aligned along the nanochain obtained for σ = ↑ (upper panel) and σ = ↓ (bottom panel). The magnetic field *B* is expressed in units of *t*/(*g*μ_B_/2).

Figure 4 shows evolution of the spin-dependent spectrum ρ*_i_*_σ_(ω) as a function of a varying magnetic field. At a critical value (*B* ≈ 0.2) we observe the emergence of zero-energy quasiparticles, whose spectral weights strongly depend on the spin σ.

**Figure 5:**
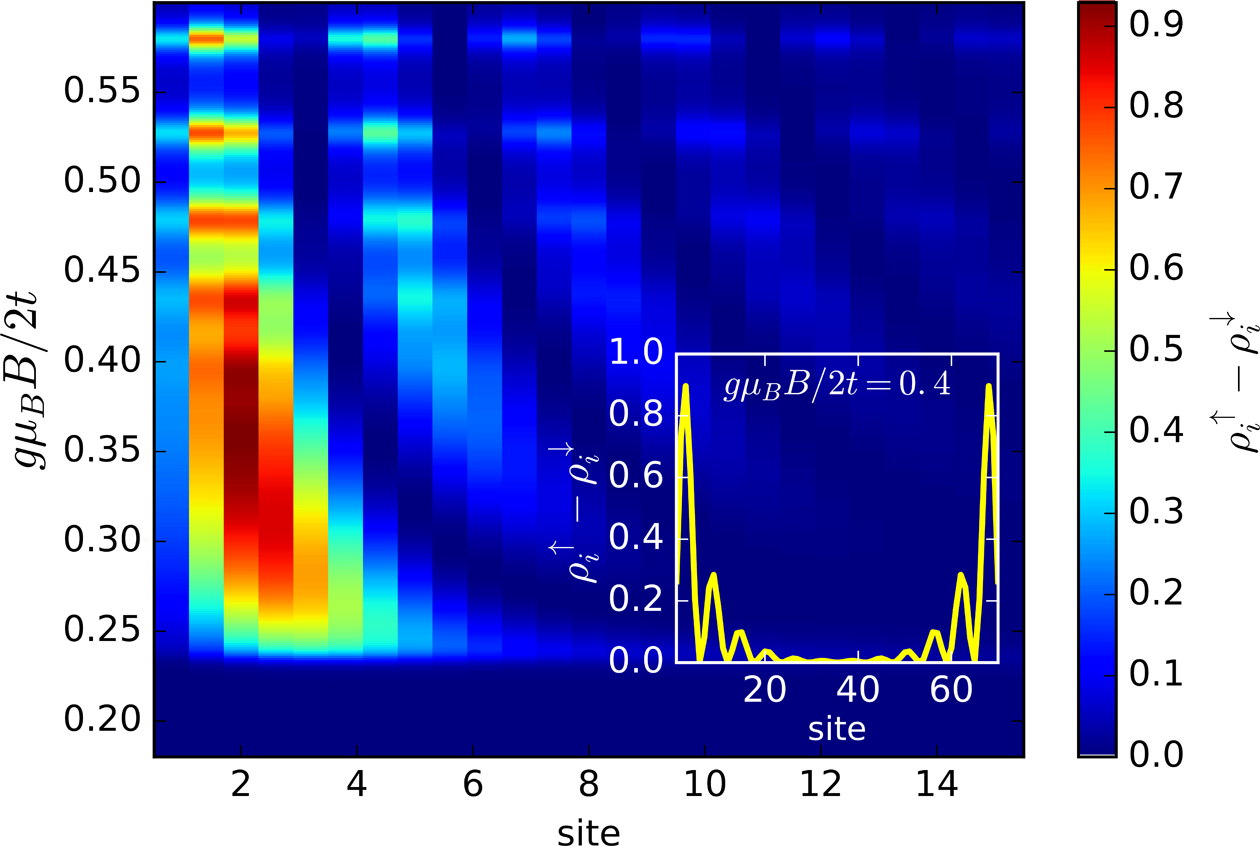
Magnetically polarized spectrum ρ*_i,↑_*(ω) − ρ*_i,↓_*(ω) obtained at ω = 0 for peripheral sites of the Rashba chain.

For a better understanding of the polarized zero-energy quasiparticles, we present in Figure 5 the spatial profiles of the zero-energy (Majorana) quasiparticles. As usually such quasiparticles emerge near the edges of a nanoscopic chain, practically over 10 to 15 sites (see inset). Note the substantial quantitative difference between these zero-energy quasiparticles appearing in ↑ and ↓ spin sectors. This “intrinsic polarization” of the Majorana modes has been previously suggested in [46], and recently we have proposed [47] their empirical detection by means of selective equal-spin Andreev reflection (SESAR) spectroscopy.

The main idea is to apply a bias voltage *V* between the STM tip and the superconducting substrate, inducing a charge transport that, in a subgap regime (
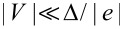
) originates from the Andreev (particle to hole) scattering mechanism. The polarized Andreev current can be expressed by the Landauer–Büttiker formula

[14]
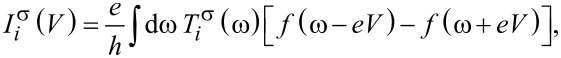


where transmittance is defined as


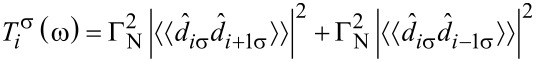


and


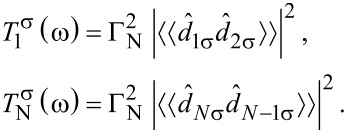


The anomalous Green’s functions can be computed numerically from the solution of the Bogoliubov–de Gennes equations of this model (Equation 10). The net spin current 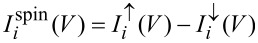 turns out to be predominantly sensitive to the Majorana end-modes. Its differential conductance 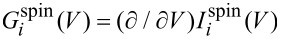 can thus distinguish the polarized Majorana quasiparticle (near *V* = 0) from the YSR states (appearing at finite voltage).

Bound states can leak to other side-attached nanoscopic objects. This proximity effect has been also predicted for the Majorana quasiparticles by E. Vernek et al. [48] and it has been indeed observed experimentally by M. T. Deng and co-authors [49]. Inspired by this achievement, extensive studies have been carried out regarding the YSR states coalescencing into the zero-energy Majorana states in side-coupled quantum dots driven by electrostatic or magnetic fields [50–52]. This issue would be particularly important when attempting to braid the Majorana end modes, e.g., in T-shape nanowires upon turning on and off the topological superconducting phase in its segments. We briefly analyse here the polarized zero-energy Majorana modes leaking into the multi-site quantum dot (comprising ten lattice sites) side-attached to the proximitized Rashba chain discussed above.

**Figure 6:**
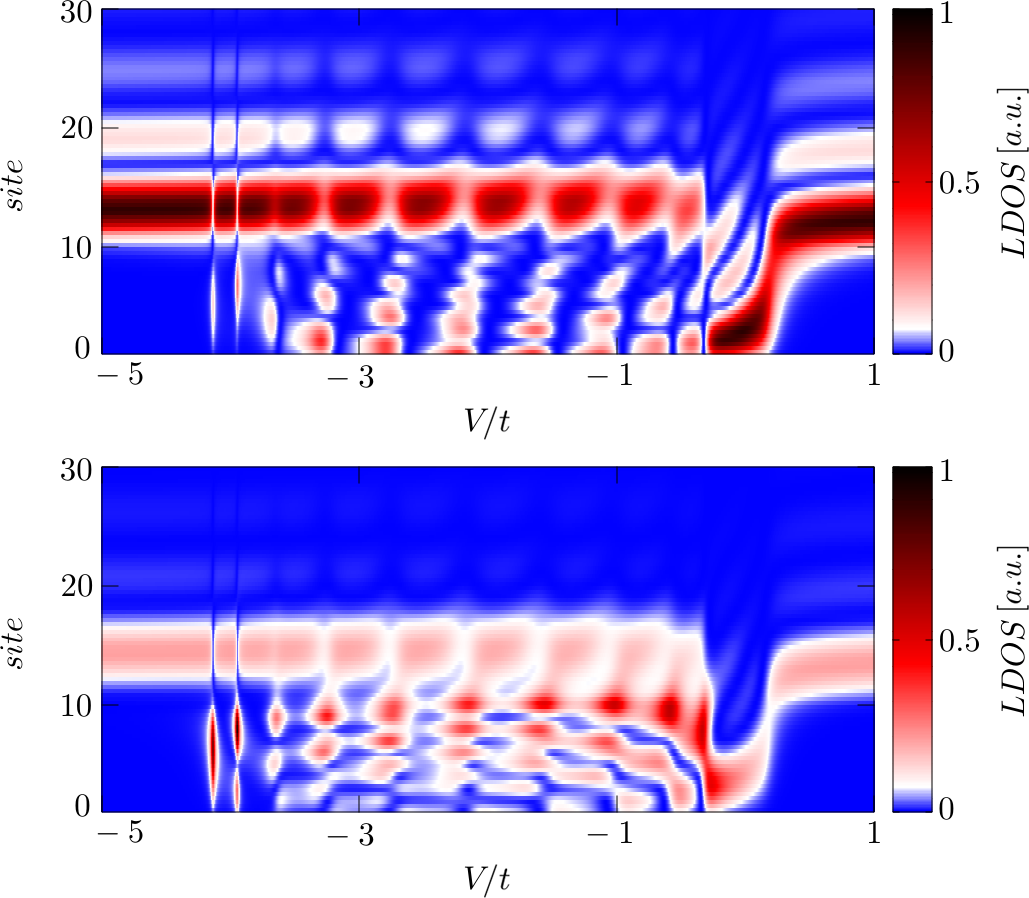
Leakage of the spin-polarized Majorana quasiparticles from the topological superconducting phase of the Rashba chain (*i* ≥ 10) onto the side-attached multi-site (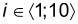) quantum dot. The upper and bottom panel show ρ*_iσ_*(ω) at ω = 0 for ↑ and ↓ spin, respectively.

Figure 6 displays the spatial profile of the polarized spectrum obtained at ω = 0 as a function of the gate voltage *V*_g_, which detunes the energies *V*_g_ = ε*_i_* − μ of the multi-site (1 ≤ *i* ≤ 10) quantum dot. For numerical calculations we used the model parameters λ = 0.15*t*, μ = −2*t*, Δ*_i_* = 0.2*t* and *B > B*_c_, which guarantee the Rashba chain to be in its topologically nontrivial superconducting phase, hosting the zero-energy Majorana quasiparticles (intensive black or red regions). We clearly observe that for some values of *V*_g_ these Majorana modes spread over the entire quantum dot region. By inspecting Figure 6 we furthermore notice the pronounced spatial oscillations of these zero-energy modes. In our opinion, this is a signature of a partial delocalization of the polarized Majorana quasiparticles. Surprisingly, this process seems to be less efficient in the minor spin (σ = ↓) section. This effect has to be taken into account, when designing nanostructures for a controllable spatial displacement of the Majorana modes (criticial for the realization of quantum computations with use of the Majorana-based qubits) either by electrostatic or magnetic means. Some proposals for such nanodevices have been recently discussed by several authors [52–53].

In summary of this section, we emphasize that the Majorana modes coalescing from the YSR states in the proximitized Rashba nanowire are characterized by their magnetic polarization. Indeed, such a feature has been recently observed by STM spectroscopy with use of a polarized tip [42]. We have studied here the evolution of the polarized quasiparticle states with respect to the magnetic field (Figure 4) and investigated the spatial oscillations of the Majorana zero-energy modes near the chain edges (Figure 5). Finally, we analyzed leakage of the polarized Majorana modes on the multi-site quantum dots, revealing their partial delocalization (Figure 6).

### Majorana vs Kondo effect

**Figure 7:**
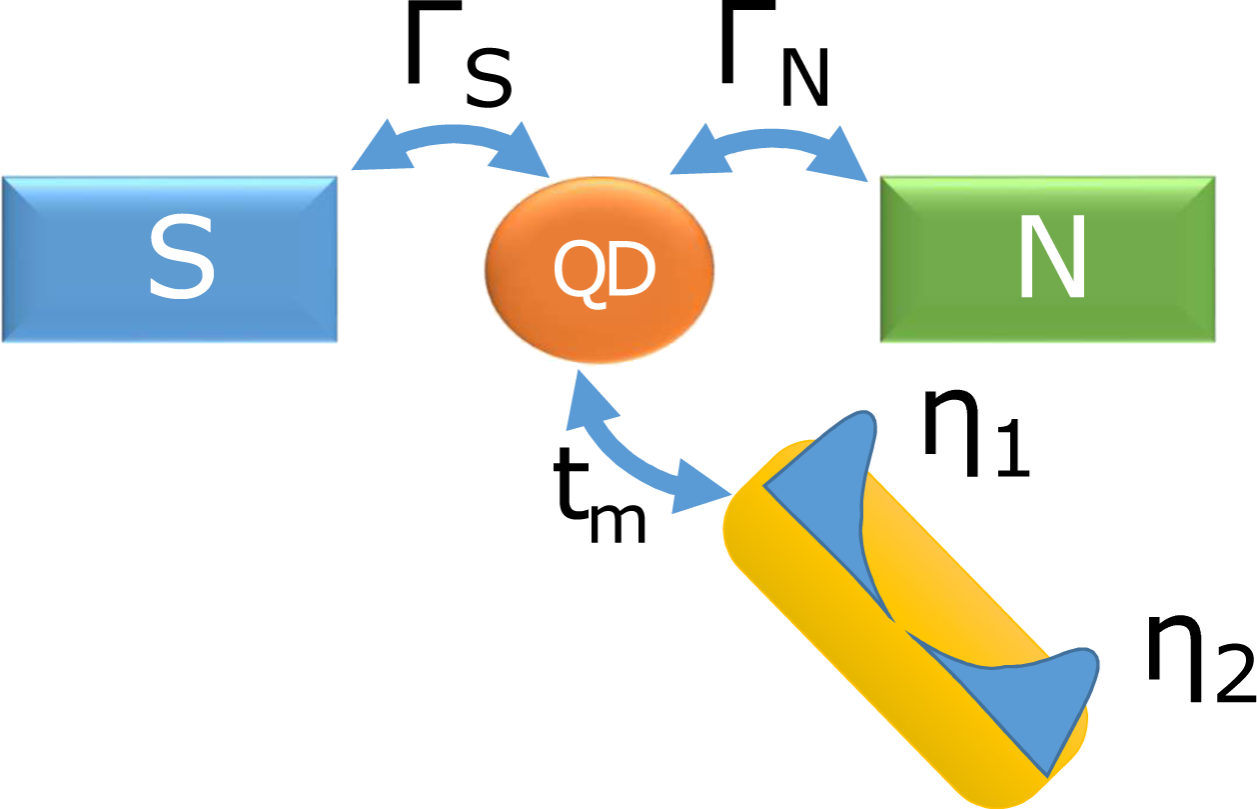
Schematic illustration of the quantum dot (QD) coupled between the metallic (N) and superconducting (S) leads and hybridized with the Rashba nanowire, hosting the Majorana quasiparticles η_1_ and η_2_ at its edges.

In previous section we have discussed the polarized Majorana modes leaking into side-attached objects, such as single impurities or segments of normal nanowires. In this section we shall focus on the correlation effects [54–56], confronting the Majorana quasiparticle with the Kondo effect (both manifested at zero energy). This can be practically achieved using STM-type configurations sketched in Figure 7. In particular, we consider the subgap Kondo effect, effectively driven by the Coulomb repulsion *U* and coupling of the quantum dot (QD) with the normal lead Γ_N_ in presence of electron pairing (induced via Γ_S_), which has a significant influence on the spin-polarized bound states of the QD. The basic mechanism of this subgap Kondo effect showing up near the quantum phase transition has been earlier considered by us in absence of the Rashba nanowire [24,57]. Our considerations can be practically verified within STM geometry [39–40] using magnetic atoms (e.g., Fe) and side-coupled nonmagnetic atoms (for instance Ag or Au) deposited on the superconducting substrate (such as Pb or Al) probed with a conducting STM tip [42].

The topological superconducting phase, hosting the Majorana modes, can be driven in semiconducting wires [58–59] or in nanochains of magnetic atoms [39–42] through nearest-neighbor equal-spin pairing. The efficiency of such *p*-wave pairing differs for each spin [47], giving rise to polarization of the Majorana quasiparticles, with noticeable preference for the ↑ sector (see Figure 4). In order to study the correlation effects we shall assume here a complete polarization of the Majorana quasiparticles. We thus focus, for simplicity, on the topological state originating from intersite pairing of only ↑ electrons and consider its interplay with the correlations. Let us remark, however, that the superconducting lead mixes both the QD spins with the side-attached Majorana quasiparticle [60]. In consequence we shall observe an interesting and spin-dependent relationship between the Majorana and Kondo states that could be probed by the polarized Andreev (particle-to-hole conversion) mechanism.

Our setup (Figure 7) can be described by the following Anderson-type Hamiltonian

[15]
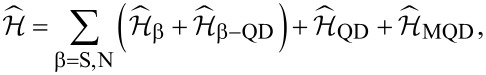


where 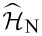 corresponds to the metallic electrode, 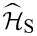 refers to the *s*-wave superconducting substrate and the correlated QD is modeled by 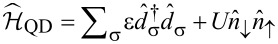, where ε denotes the energy level and *U* stands for the repulsive interaction between opposite spin electrons. The QD is coupled to both β = N,S reservoirs through 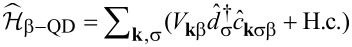 and we assume a wide bandwidth limit, using the constant couplings Γ_β_. It can be shown [61–64] that for energies 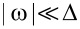 the super-conducting electrode induces the static on-dot pairing


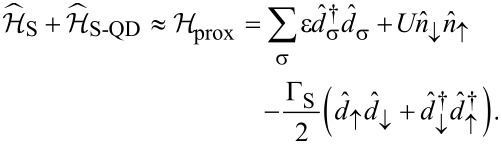


Taking into account the finite magnitude of superconducting gap [50] does not affect the main conclusions of our study.

The effective Majorana modes of the nanowire can be modeled by [65]


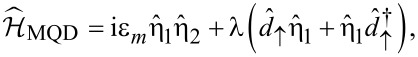


where 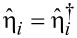 are Hermitian operators and ε*_m_* corresponds to an overlap between Majoranas. We recast these operators by the standard fermionic ones [66] 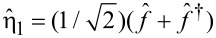 and 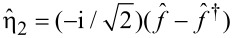. Finally, the Hamiltonian of Equation 15 simplifies to

[16]
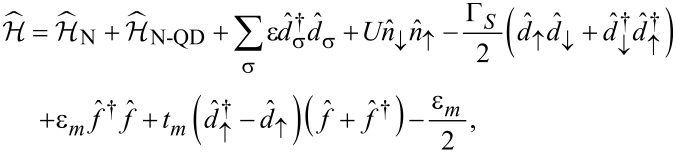


with the auxiliary coupling 
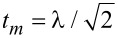
. The subgap Kondo physics originates in this model from the Coulomb term 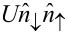 and the effective spin-exchange interactions due to 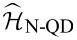. It has been shown [23–24] that under specific conditions the on-dot pairing can cooperate with the subgap Kondo effect. This particular situation occurs only near the quantum phase transition.

Let us examine how the subgap Kondo effect gets along with the Majorana mode. Earlier studies of the correlated quantum dot coupled to both normal (conducting) electrodes indicated that the side-attached Rashba chain leads to a competition between the Kondo and Majorana states [67–72]. For a sufficiently long wire (ε*_m_* = 0) the Kondo effect persists only in the spin-channel ↓, whereas for ↑ electrons there appears a dip in the spectral density at ω = 0. The resulting tunneling conductance is then partly reduced (from the perfect value 2*e*^2^/*h*) to the fractional value 3*e*^2^/2*h* [67–68,71–73]. In contrast, for the short Rashba wires (with ε*_m_* ≠ 0) the Kondo physics persists in both spin channels.

In our present setup (Figure 7) the correlated quantum dot is between the metallic and superconducting reservoirs, therefore the Kondo effect is additionally affected by on-dot pairing. Its influence is mainly controlled by the ratio *U*/Γ_S_ and partly by the level ε, determining whether the QD ground state is in the spinful or spinless configuration [23–24,62,64,74]. Obviously the latter one cannot be screened. For instance, for the half-filled QD (ε = −*U*/2) the spinful (doublet) configuration occurs in the regime *U* ≥ Γ_S_.

**Figure 8:**
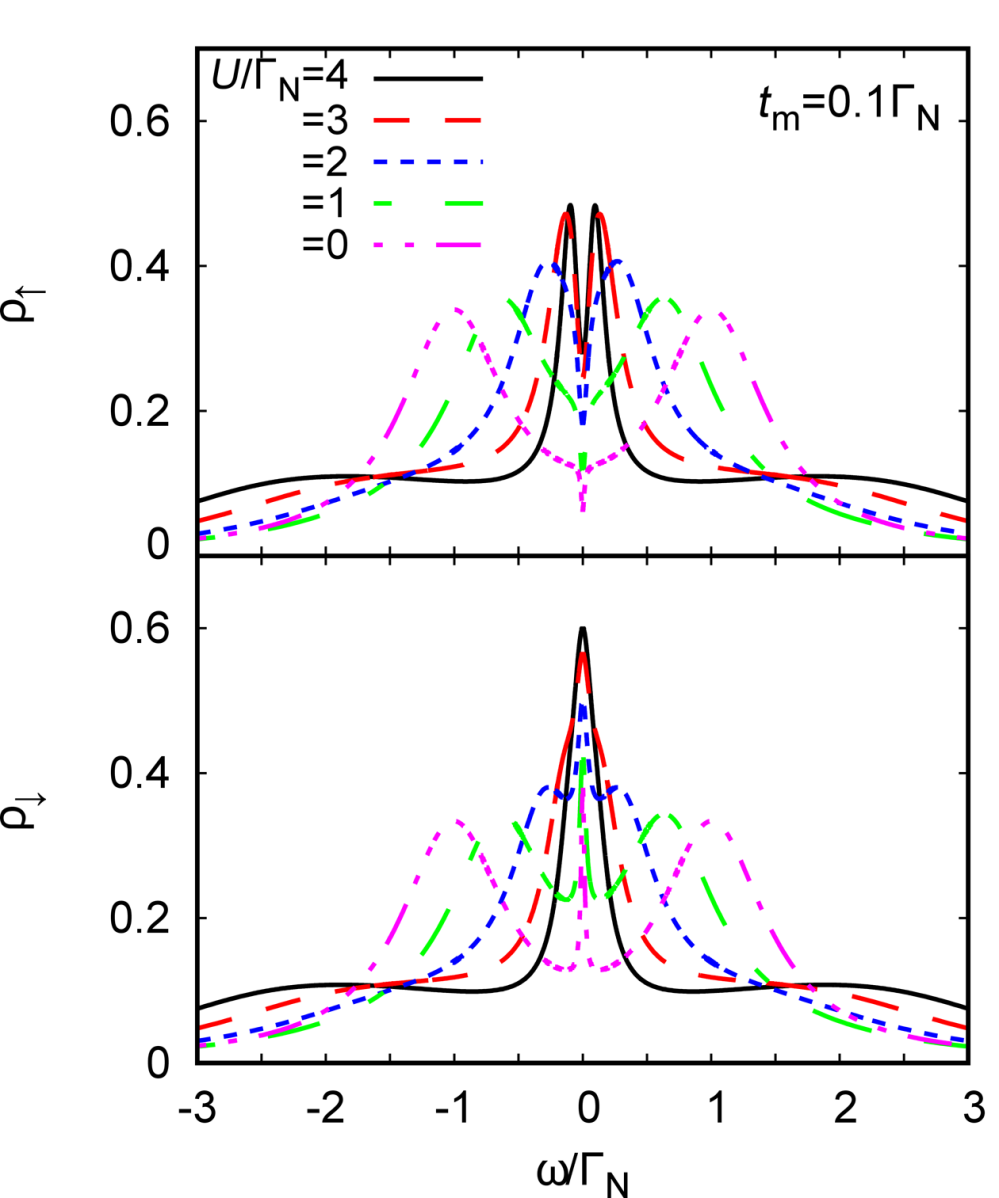
The polarized spectral function ρ_σ_(ω) obtained at zero temperature for the half-filled QD (ε = −*U*/2), Γ*_S_* = 2Γ*_N_*, *t_m_* = 0.1Γ_N_ and several values of the Coulomb potential *U* (as indicated). Energies are expressed in units of Γ_N_.

For studying the correlations we adopt perturbative treatment of the Coulomb potential, treating it self-consistently to the second order in the normal and anomalous channels [62,75]. Specific expressions have been provided by us in [24]. Figure 8 shows the spectral function ρ_σ_(ω) for both spins obtained at zero temperature for the Coulomb potential *U*, covering the (spinless) singlet and (spinful) doublet configurations. In the weak interaction regime we observe appearance of two YSR states. For *U* ≈ Γ_S_ these peaks merge, signaling the quantum phase transition. The Kondo effect shows up only in the correlated limit (*U >* Γ_S_), but its spectroscopic signatures are qualitatively different for each of the spins. Leakage of the Majorana quasiparticle suppresses the low-energy states of ↑ electrons. We notice that the initial density (for *t_m_* = 0) is reduced by half, whereas we observe a constructive influence of the Majorana quasiparticle on opposite-spin ↓ electrons.

**Figure 9:**
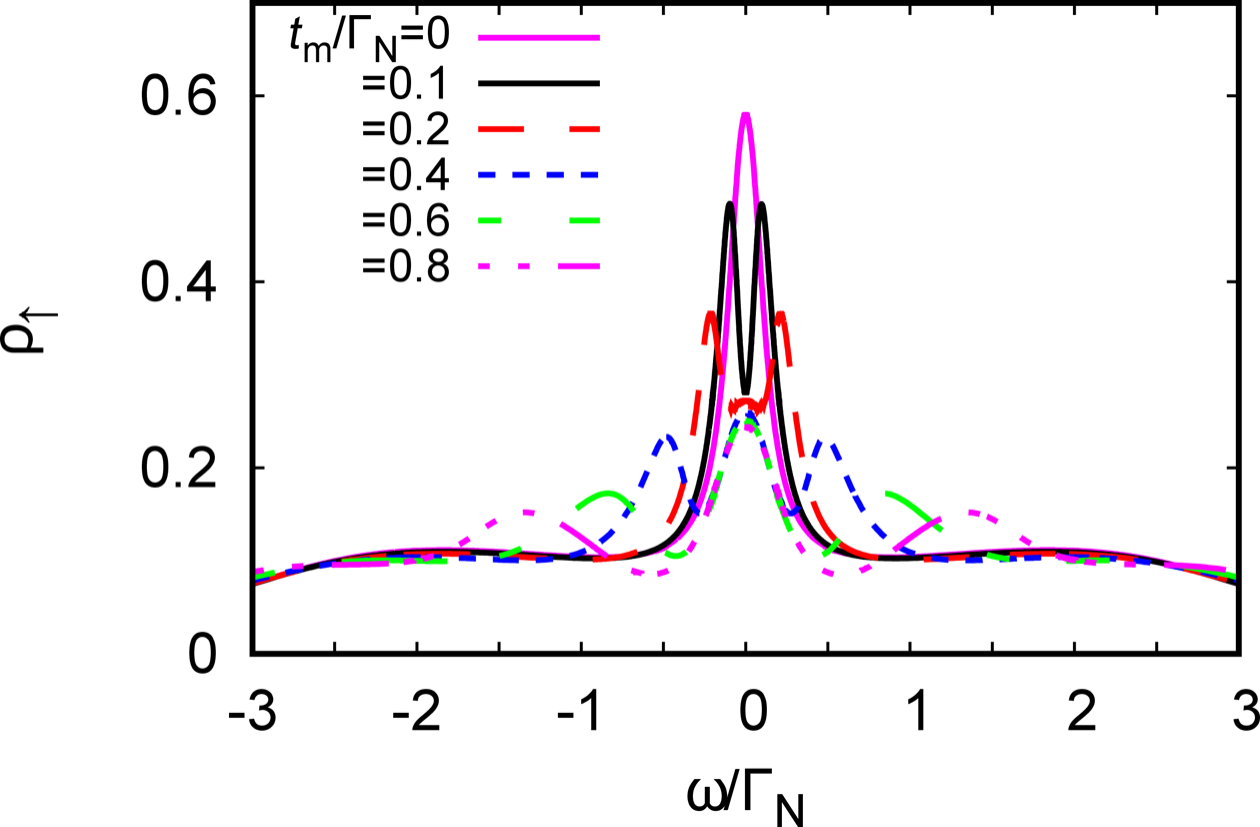
The spectral function ρ_↑_(ω) of the half-filled quantum dot (ε = −*U*/2) obtained at *T* = 0 for Γ*_S_*/Γ_N_ = 2, *U*/Γ_N_ = 4 and several values of *t_m_* (as indicated).

Figure 9 shows evolution of the spectral function ρ_↑_(ω) for various couplings *t_m_*. In the weak-coupling limit we clearly observe a reduction (by half) of the initial density of states. With increasing *t_m_* the spectrum develops the three-peak structure that is typical for the “molecular” limit. This behavior indicates that the Majorana and Kondo states have rather a complicated relation, which is neither competitive nor cooperative. In fact, some novel scaling laws have been recently reported by several authors [69–70,76–79] also considering the correlation effects directly in the Rashba nanowire.

## Conclusion

We have studied the polarized bound states of magnetic impurities embedded in an *s*-wave superconducting material, taking into account the spin–orbit and/or Coulomb interactions. We have shown that spin–orbit coupling strongly affects the subgap states, both of the single impurities and their conglomerate arranged into a nanoscopic chain. For the case of single magnetic impurity the spin–orbit interaction (i) shifts the quantum phase transition towards higher magnetic coupling *J*_c_, (ii) enhances the spatial size of the YSR states, and (iii) smoothes the particle–hole oscillations. For the magnetic chain spin–orbit coupling combined with the Zeeman term induce the topologically nontrivial superconducting state and indirectly give rise to substantial polarization of the Majorana modes (Figure 4), the oscillations of which show up near the chain edges (Figure 5). The polarized Majorana quasiparticles can also leak into other side-coupled objects, such as single or multiple quantum impurities (Figure 6). These polarized Majorana quasiparticles can be controlled by a magnetic field or by an electrostatic potential. This would be important for future quantum computers using qubits based on topologically protected Majorana states. Finally, we have also confronted the Majorana quasiparticles with the subgap Kondo effect, revealing their complex relationship that can be hardly regarded as competitive or collaborative in some analogy to the Kondo effect originating from multiple degrees of freedom [80]. The aforementioned spin-polarized effects can be experimentally verified by polarized ballistic tunneling or by using STM spectroscopy, relying on the selective equal-spin Andreev reflections.
